# Why are we willing to tolerate manipulation? Love addiction and perceived acceptability of gaslighting: the mediating effects of sense of giving and relationship power

**DOI:** 10.3389/fpsyg.2025.1525402

**Published:** 2025-04-16

**Authors:** Yuxiao Wang, Jinjin Li, Yu Zhang, Xiangcai He, Yu Luo

**Affiliations:** ^1^School of Psychology, Guizhou Normal University, Guiyang, China; ^2^Key Laboratory of Brain Function and Brain Disease Prevention and Treatment of Guizhou Province, Guiyang, China; ^3^School of Psychology, Chengdu Medical College, Chengdu, China

**Keywords:** love addiction, sense of giving, relationship power, gaslighting, chain mediation, Chinese university students

## Abstract

**Objective:**

This study investigates how love addiction influences individuals’ perceived acceptability of gaslighting in romantic relationships, focusing on the mediating roles of sense of giving and relationship power.

**Methods:**

Surveys were administered online to university students in Southwestern China between October and December 2023, utilizing the Love Addiction Inventory, Sense of Giving Questionnaire, Sexual Relationship Power Scale, and Gaslighting Questionnaire. From an initial pool of 480 responses, 464 valid questionnaires were retained for analysis (96.7% valid response rate). Data were processed and analyzed using SPSS 27.0, beginning with descriptive statistics (means, standard deviations), followed by Pearson correlation analysis and chain mediation analysis.

**Results:**

The findings revealed that love addiction did not directly predict perceived acceptability of gaslighting (*β* = 0.037, *p* = 0.549) after accounting for the mediating roles of sense of giving and relationship power. Instead, love addiction influenced perceived acceptability of gaslighting entirely through three pathways: (1) an independent mediating effect via sense of giving (*β* = 0.106, 95% CI [0.014,0.202], 36.30% of total effect), (2) an independent mediating effect via relationship power (*β* = 0.106, 95% CI [0.054,0.166], 36.30% of total effect), and (3) a chain mediation through both sense of giving and relationship power (*β* = 0.043, 95% CI [0.009,0.079], 14.73% of total effect). Together, these mediators explained 87.33% of the total effect, indicating a complete mediation model.

**Conclusion:**

These results emphasize the importance of considering the effects of love addiction when understanding perceived acceptability of gaslighting and highlight that sense of giving and relationship power can explain the potential mechanisms of the association between love addiction and perceived acceptability of gaslighting. This provides valuable insights for developing interventions aimed at mitigating gaslighting.

## 1 Introduction

In recent years, gaslighting has received increasing attention for its negative impact on the psychological and emotional health of victims (Sweet, 2019; March et al., 2023). Research has shown that more than half of individuals in romantic relationships report having been subjected to gaslighting by their partners (He et al., 2012; Sweet, 2019; Spencer et al., 2021; Leemis et al., 2022). While gaslighting is commonly reported in romantic relationships, it is essential to clarify that not all behaviors labeled as “gaslighting” meet the criteria for severe, intentional manipulation. True gaslighting is a covert form of psychological and emotional abuse. It systematically distorts victims’ perception of reality by eroding their trust in personal memories and judgments. This is achieved through gradual manipulation tactics, including denial, misinformation, and lying (Sweet, 2019; Hailes and Goodman, 2023; March et al., 2023). In the meantime, it is critical to distinguish between gaslighting as a form of perpetration and its subjective acceptance. While gaslighting typically involves manipulative acts initiated by a perpetrator, this study focuses on individuals’ perceived acceptability of such behaviors. Specifically, their cognitive and emotional tolerance when facing a partner’s gaslighting tactics (e.g., dismissing their experiences or denying factual events). This distinction is crucial because the perceived acceptability of gaslighting may normalize psychological harm and increase vulnerability to long-term relational dysfunction. In other words, the higher an individual accepts for their partner’s gaslighting behavior, the more likely they are to become a victim of gaslighting (Lazarus, 1991; Sweet, 2019; Hailes and Goodman, 2023). If individuals experience gaslighting over a long period, it can significantly impair their cognitive abilities, self-esteem, and interpersonal relationships, with far-reaching negative effects on well-being (Anderson et al., 2003; Stern, 2018; Sweet, 2019; Kukreja and Pandey, 2023). The negative effects of gaslighting on the university student population are of particular concern. Since university students are at an important stage of psychological development and socialization, their romantic relationships are exploratory and unstable (Arnett, 2000; Schwartz et al., 2013; Shulman and Connolly, 2013). This makes them more vulnerable to gaslighting (Spencer et al., 2021; Neilson et al., 2023; Carone et al., 2024). Therefore, understanding the factors that influence perceived acceptability of gaslighting in university students is critical to promoting positive development and fostering healthy romantic relationships.

Among the various risk factors that contribute to gaslighting, love addiction may play a significant role. Driven by specific risk factors, it can lead to serious impairments in personal functioning and well-being (Bockmann and Sanches, 2022). Love addiction comprises maladaptive, pervasive, and excessive interest in romantic partners, leading to impaired self-control and reduced engagement in social, professional, and leisure activities (Sussman, 2010; Earp et al., 2017). Love addicts lack adaptive self-regulation strategies. Despite being aware of the inherent problems in their romantic relationships, they still persist in their interactions with their partners. They believe that love can only be obtained through giving, suffering, and sacrifice (Kwee, 2007; Sanches and John, 2019; Bockmann and Sanches, 2022). This causes love addicts to rationalize their partner’s misbehavior in the relationship as necessary to maintain the relationship. This cognitive distortion impairs their ability to recognize manipulation, thereby increasing tolerance for gaslighting (Sussman, 2010; Bonache et al., 2019; Redcay and McMahon, 2021). At the same time, individuals who are addicted to love are more likely to prioritize maintaining their relationships over their well-being when relationship interests and self-interests conflict (Kwee, 2007; Gori et al., 2023). Even in an unhealthy relationship, they may consciously or unconsciously ignore signs of relationship dysfunction to gain emotional support from their partner. This makes it difficult for them to disengage from harmful relationship dynamics, which may lead to a range of negative consequences (Sussman, 2010; Earp et al., 2017; Sanches and John, 2019). Therefore, it is logical to hypothesize that love addiction positively predicts perceived acceptability of gaslighting.

In romantic relationships, individuals usually invest resources such as time, energy, and finances to foster a connection with each other (Goodfriend and Agnew, 2008). In addition to the tangible and intangible investments needed to sustain a relationship, the sense of giving plays an equally important role (Day and Impett, 2018; Zhou, 2018; Jiang et al., 2021; Tang et al., 2024). Sense of giving refers to an individual’s perception of actively providing for their partner, encompassing their awareness of personal contributions to the relationship, emotional investment, and subjective feelings resulting from the act of giving (Zhou, 2018; Jiang et al., 2021). Although partners are interdependent agents with shared emotional bonds and altruistic motivations, conflicts between individual needs and relational goals inevitably emerge in long-term commitments, rendering the balance of personal and dyadic priorities a critical challenge (Maner and Gailliot, 2007; Day and Impett, 2016). Individuals who are addicted to love are more likely to prioritize their partner’s needs in order to maintain relationship harmony, and they prove their worth by over-giving (Sussman, 2010; Markus and Kitayama, 2014; Mattingly et al., 2014). The resulting great sense of giving may exacerbate relationship imbalance and may also lead to individuals becoming increasingly willing to sacrifice personal boundaries to maintain relationships. In the process, the cost of leaving the relationship increases over time as the emotional and psychological investment also increases. As a result, individuals may become more tolerant even in the face of a partner’s abusive behavior when the potential for relationship breakdown exists (Festinger, 1957; Goodfriend and Agnew, 2008; Xiang et al., 2017). Therefore, we propose that the link between love addiction and perceived acceptability of gaslighting is mediated by sense of giving. Specifically, love addiction is associated with a high sense of giving, which in turn is linked to perceived acceptability of gaslighting.

Current research suggests that relationship power imbalances are a key factor in the occurrence of gaslighting (Sweet, 2019; Hailes and Goodman, 2023). Relationship power is the capacity of an individual to exert influence over the thoughts, feelings, and behaviors of another by controlling decision-making and relationship dynamics (Simpson et al., 2015; Martín-Lanas et al., 2021). Gaslighting can be extended to include a single behavior or series of behaviors performed by anyone in a high-power position to manipulate low-power others to induce doubt in their cognitive faculties or recollection of events. Over time, such dynamics increase the victim’s vulnerability to control (Davis and Ernst, 2020; Tobias and Joseph, 2020). Love addicts may have low power roles in romantic relationships, usually due to emotional dependence, fear of losing their partner, and beliefs about love (Sussman, 2010; Giacobbe et al., 2024; Rogier et al., 2024). This also makes it difficult for them to challenge their partner’s authority or question their partner’s abusive behavior, increasing the power imbalance and making them more vulnerable to gaslighting (Drigotas et al., 1999; Sussman, 2010).

Consequently, love addiction exacerbates the power imbalance in the romantic relationship, a dynamic that may make it increasingly difficult for love addicts to challenge or confront gaslighting. Thus, we suggest that relationship power mediates the relationship between love addiction and perceived acceptability of gaslighting.

According to the interdependence theory, individuals in a romantic relationship influence each other’s behavior and decision-making, creating complex patterns of interaction that determine the outcome of the relationship (Kelly, 1987). Within this framework, the sense of giving and relationship power are closely related, and over-giving may diminish one’s relationship power. For example, those who consistently prioritize their partner’s needs over their tend to relinquish control in the relationship, either intentionally or unintentionally placing themselves in a more passive and vulnerable role (Liu, 2017). For love addicts, their desires and beliefs about romantic relationships can cause them to invest more in them, which in turn may diminish their power in the relationship (Reynaud et al., 2010; Liu, 2017; Sanches and John, 2019). This dynamic makes them more vulnerable to gaslighting. Building on these mechanisms, we propose that love addiction amplifies perceived acceptability of gaslighting through elevating the sense of giving, then diminishing relationship power.

The proposed chain mediation model integrates social exchange theory (Blau, 2017) and interdependence theory (Kelly, 1987) to explain how love addiction fosters perceived acceptability of gaslighting through relational dynamics. According to social exchange principles, love addicts’ hyper-investment and excessive giving driven by abandonment anxiety disrupt reciprocity norms (Sussman, 2010). This creates a debt dynamic (Xiang et al., 2017), where individuals rationalize tolerating manipulation to justify their sacrifices, elevating sense of giving as a cognitive gateway to gaslighting acceptance. Simultaneously, interdependence theory clarifies how such imbalanced investments erode relationship power: prioritizing a partner’s needs systematically diminishes autonomy (Liu, 2017), while emotional dependence lowers the perceived viability of alternatives (Rusbult and Van Lange, 2003), trapping individuals in passive roles where challenging manipulative behaviors risks catastrophic relational dissolution (Drigotas et al., 1999). Critically, these mechanisms intersect—excessive giving (social exchange violation) precipitates power loss (interdependence imbalance), forming a relational cascade that entrenches acceptability to gaslighting.

To summarize, this study constructed a chain mediating model to clarify the impact of love addiction on perceived acceptability of gaslighting. Based on existing theoretical and empirical studies, this study hypothesized that (1) love addiction significantly and positively predicts perceived acceptability of gaslighting, (2) sense of giving mediates the relationship between love addiction and perceived acceptability of gaslighting, (3) relationship power mediates the relationship between love addiction and perceived acceptability of gaslighting, and (4) sense of giving and relationship power play a chain mediating role between love addiction and perceived acceptability of gaslighting.

## 2 Materials and methods

### 2.1 Participants

This study received ethical approval from the University Ethics Committee of the author’s institution. Participants were recruited online through WeChat and WJX platform from universities in Southwestern China between October and December 2023. All participants provided informed consent prior to participation. From an initial pool of 480 respondents, 16 cases were excluded due to patterned responding (e.g., straight-lining). The remaining 464 participants (96.7% valid response rate) met the eligibility criteria (e.g., in a romantic relationship for at least 6 months) and were retained for analysis. Participants were aged 18–30 years (*M* = 22.29, *SD* = 2.13), with no significant age differences between males (*M* = 22.12, *SD* = 2.20) and females (*M* = 22.38, *SD* = 2.09), *t* = 1.220, *p* = 0.223. The sample included 163 males (35.1%) and 301 females (64.9%), comprising 316 undergraduates (68.1%) and 148 postgraduates (31.9%).

### 2.2 Measures

#### 2.2.1 Love addiction inventory

The Love Addiction Inventory was developed by Costa et al. (2021). It consists of 24 items, covering six dimensions: Salience, Withdrawal, Tolerance, Mood Modification, Relapse, and Conflict. It employs a 5-point Likert scoring system, where “1” indicates “Never” and “5” indicates “Always,” with higher scores reflecting greater levels of love addiction. For this study, the scale was translated into Chinese following a standard forward- and back-translation procedure. First, two bilingual psychologists independently translated the original English items into Chinese. Discrepancies were resolved through discussion, and a third translator back-translated the Chinese version to ensure conceptual equivalence with the original scale. The Chinese adaptation was then pilot-tested with a small sample (n = 30) of university students to confirm comprehension and cultural appropriateness. The Cronbach’s *α* coefficient for this scale in this study was 0.937. The structural validity of the scale was also acceptable (*χ^2^/df* = 2.954, RMSEA = 0.065, CFI = 0.943, TLI = 0.929, SRMR = 0.061).

#### 2.2.2 Sense of giving questionnaire

The Sense of Giving Questionnaire was developed by Jiang et al. (2021). It consists of 14 items, divided into three dimensions: Cognitive Giving, Emotional Giving, and Behavioral Giving. It uses a 7-point Likert scoring system, where “1” denotes “Strongly Disagree” and “7” denotes “Strongly Agree,” with higher scores indicating a greater sense of giving. As the original scale was developed in Chinese by Jiang et al. (2021), no additional translation was required for this study. The Cronbach’s *α* coefficient for this scale in this study was 0.884. The structural validity of the scale was also acceptable (*χ^2^/df* = 2.813, RMSEA = 0.063, CFI = 0.956, TLI = 0.937, SRMR = 0.044).

#### 2.2.3 Sexual relationship power scale

The Sexual Relationship Power Scale was originally developed by Pulerwitz et al. (2000). The Chinese version used in this study was adapted by Zhang (2018), followed by validation with a Chinese sample (Zhang, 2018). The scale includes two subscales: Relationship Control and Decision-Making Dominance. Previous studies have shown that the Relationship Control subscale has higher reliability, whereas the Decision-Making Dominance subscale exhibits relatively weaker psychometric properties in most populations and settings (McMahon et al., 2015). Therefore, this study utilized only the Relationship Control subscale to assess relationship power. Given that the research does not involve sexual behavior and considering participants who may not have engaged in sexual activities, items related to sexual behavior were excluded. The revised subscale, comprising 11 items not related to sexual behavior, also demonstrates reliability (Pulerwitz et al., 2000). It uses a 4-point Likert scale, where “1” denotes “Strongly Agree” and “4” denotes “Strongly Disagree,” with higher scores indicating greater power in the relationship. The Cronbach’s *α* coefficient for this subscale in the current study was 0.799. Structural validity was assessed via confirmatory factor analysis, showing acceptable fit (*χ^2^/df* = 2.276, RMSEA = 0.052, CFI = 0.963, TLI = 0.942, SRMR = 0.037).

#### 2.2.4 Gaslighting questionnaire

The Gaslighting Questionnaire was developed by March et al. (2023). Given the potential bias associated with directly assessing violent behaviors in intimate relationships (Ferrer-Perez et al., 2020), it is more reasonable to indirectly assess gaslighting by evaluating the perceived acceptability of gaslighting tactics (Spencer et al., 2021; March et al., 2023). The scale comprises 10 items, using a 7-point Likert scale where “1” represents “Unacceptable” and “7” represents “Acceptable,” with higher scores indicating greater perceived acceptability of gaslighting by the individual. For this study, the scale was translated into Chinese using a forward- and back-translation process. Two independent translators converted the English items into Chinese, and discrepancies were resolved through consensus. A third translator back-translated the Chinese version into English to verify accuracy. To tailor the scale for measuring perceived acceptability of gaslighting in romantic relationships, items were adapted from statements such as “A accuses B of lying...” to “Your partner accuses you of lying....” The adapted Chinese version was pilot-tested with 25 university students to ensure cultural relevance and item clarity. The Cronbach’s α coefficient for this scale in the current study was 0.906. The structural validity of the scale was also acceptable (*χ^2^/df* = 2.894, RMSEA = 0.064, CFI = 0.981, TLI = 0.966, SRMR = 0.030).

### 2.3 Statistical approach

The statistical analysis was conducted using SPSS 27.0. Initially, Harman’s single-factor test was performed to assess common method bias. Then, descriptive statistics (means, standard deviations) and Pearson correlation analyses were conducted to examine the relationships among love addiction, sense of giving, relationship power, and perceived acceptability of gaslighting. Finally, to test the hypothesized chain mediation model (love addiction → sense of giving → relationship power → perceived acceptability of gaslighting), Model 6 from PROCESS macro was employed with 5,000 bootstrap resamples to estimate 95% bias-corrected confidence intervals (CIs) for the indirect effects, enhancing the robustness of parameter estimates. A *p*-value < 0.05 was considered statistically significant.

## 3 Results

### 3.1 Common method biases

The results of the Harman single-factor test showed that there were 13 factors with eigenvalues greater than 1, and the variance explained by the largest factor was 26.238% (less than 40%). Therefore, no significant common method bias was detected (Podsakoff et al., 2003).

### 3.2 Descriptive statistics and correlation analysis

Table 1 presents the descriptive statistics and correlation analysis of the research variables. The results indicate that love addiction is significantly and positively correlated with both sense of giving and perceived acceptability of gaslighting, while negatively correlated with relationship power. Moreover, sense of giving is positively correlated with perceived acceptability of gaslighting and negatively correlated with relationship power. Last, relationship power demonstrates a significant negative correlation with perceived acceptability of gaslighting.

**Table 1:** Mean, standard deviation, and correlation matrix of each variable (*N* = 464).

Variables	*M (SD)*	1	2	3	4
1. Love addiction	2.69 (0.64)	1			
2. Sense of giving	4.25 (1.10)	0.690**	1		
3. Relationship power	3.10 (0.48)	−0.369**	−0.349**	1	
4. Perceived acceptability of gaslighting	1.81 (0.93)	0.279**	0.301**	−0.441**	1

*p < 0.05, **p < 0.01.

### 3.3 Love addiction and perceived acceptability of gaslighting: chain mediating effect test

**Figure 1:**
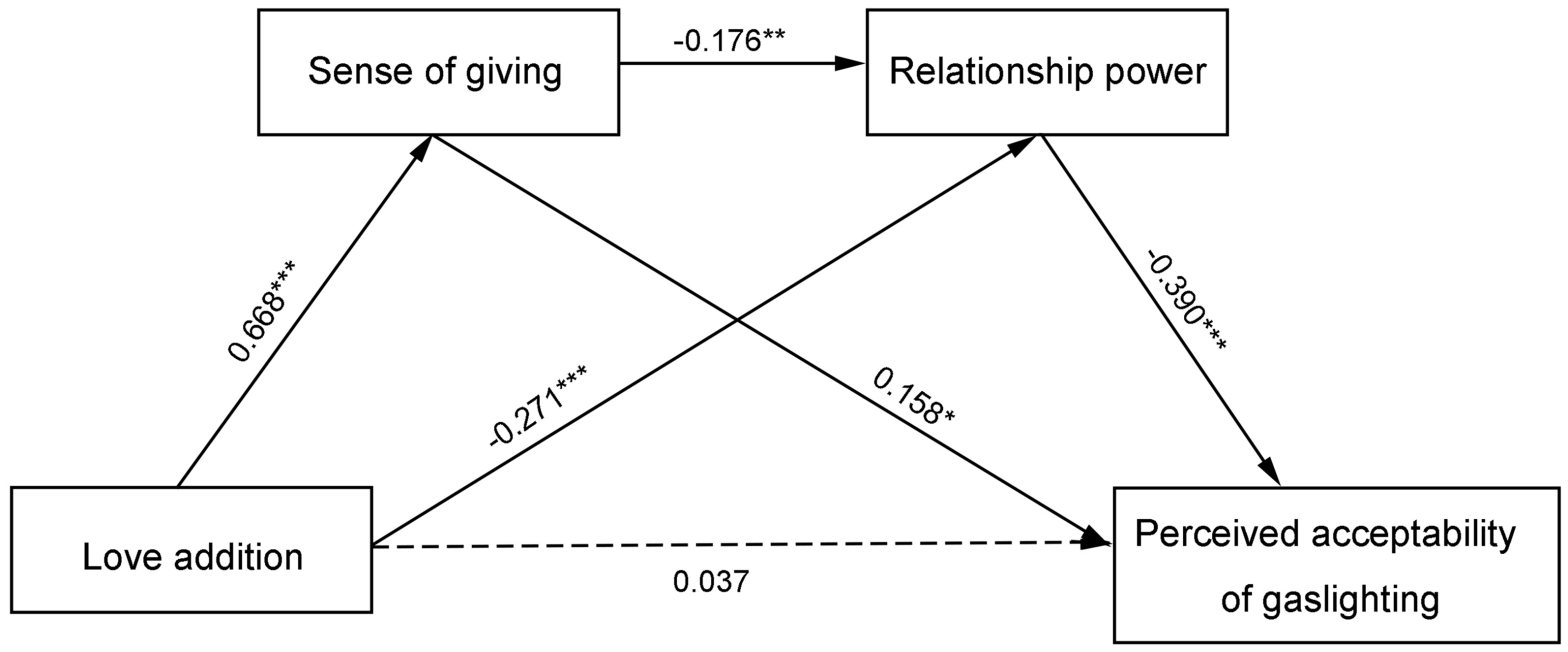
The chain mediation model of the relationship between love addiction and perceived acceptability of gaslighting. *p < 0.05, **p < 0.01, ***p < 0.001.

After controlling for the demographic variables of gender and age, a chain mediation model was tested, which consisted of three indirect effects as follows: (1) love addiction promotes perceived acceptability of gaslighting via sense of giving, (2) love addiction promotes perceived acceptability of gaslighting via relationship power, and (3) love addiction promotes perceived acceptability of gaslighting via sense of giving and relationship power (Figure 1). The stepwise regression analysis is shown in Table 2. The results suggested a positive effect of love addiction on sense of giving (*β* = 0.668, *t* = 20.778, *p* < 0.001), and a negative effect of love addiction on relationship power (*β* = −0.271, *t* = −4.477, *p* < 0.001). A negative relationship between sense of giving and relationship power was also identified (*β* = −0.176, *t* = −2.649, *p* < 0.01). Moreover, sense of giving significantly predicted perceived acceptability of gaslighting (*β* = 0.158, *t* = 2.513, *p* < 0.05). Relationship power significantly predicted perceived acceptability of gaslighting (*β* = −0.390, *t* = −8.431, *p* < 0.001). After controlling the effects of sense of giving and relationship power, the direct effect of love addiction on perceived acceptability of gaslighting was not significant (*β* = 0.037, *t* = 0.601, *p* = 0.549).

**Table 2:** Results of regression analysis (*N* = 464).

Predictive variable	Model1: sense of giving		Model 2: relationship power		Model 3: perceived acceptability of gaslighting	
	*β*	*t*	*β*	*t*	*β*	*t*
Gender	0.189	2.884**	−0.344	−3.849***	−0.092	−1.024
Age	0.027	1.811	0.021	1.045	−0.004	−0.186
Love addiction	0.668	20.778***	−0.271	−4.477***	0.037	0.601
Sense of giving			−0.176	−2.649**	0.158	2.513*
Relationship power					−0.390	−8.431***
*R^2^*	0.489		0.182		0.222	
*F*	146.601		25.597		26.112	

*p < 0.05, **p < 0.01, ***p < 0.001.

Furthermore, the results of the mediating effect analysis of love addiction and perceived acceptability of gaslighting in Table 3 showed that the total effect of love addiction and perceived acceptability of gaslighting was significant (*β* = 0.292, *SE* = 0.047, 95%CI [0.200,0.384]). However, after introducing the mediators, the direct effect was not significant (*β* = 0.037, *SE* = 0.061, 95%CI [−0.084,0.157]). Specifically, the indirect effect of love addiction on perceived acceptability of gaslighting through sense of giving was significant (*β* = 0.106, *SE* = 0.047, 95%CI [0.014, 0.202]). The mediation effect (love addiction → sense of giving → perceived acceptability of gaslighting) accounted for 36.30% of the total effect. Then, relationship power mediated the relationship between love addiction and perceived acceptability of gaslighting (*β* = 0.106, *SE* = 0.029, 95%CI [0.054, 0.166]). The mediation effect (love addiction → relationship power → perceived acceptability of gaslighting) accounted for 36.30% of the total effect. Finally, the indirect effect of love addiction on perceived acceptability of gaslighting through sense of giving and relationship power was also significant (*β* = 0.043, *SE* = 0.018, 95%CI [0.009, 0.079]). The chain mediation effect accounted for 14.73% of the total effect. Since 0 is not contained in the Bootstrap 95% confidence intervals, these three indirect effects are statistically significant.

**Table 3:** Mediating effect analysis of love addiction and perceived acceptability of gaslighting (*N* = 464).

Model pathways	Effect	Boot SE	Bootstrap 95%CI	Effect ratio
Total effect	0.292	0.047	[0.200, 0.384]	100%
Direct effect	0.037	0.061	[−0.084, 0.157]	12.67%
Total indirect effect	0.255	0.054	[0.149, 0.366]	87.33%
1 → 2 → 4	0.106	0.047	[0.014, 0.202]	36.30%
1 → 3 → 4	0.106	0.029	[0.054, 0.166]	36.30%
1 → 2 → 3 → 4	0.043	0.018	[0.009, 0.079]	14.73%

1, love addiction; 2, sense of giving; 3, relationship power; 4, perceived acceptability of gaslighting.

## 4 Discussion

This study examined the love addiction and perceived acceptability of gaslighting focused on the mediating mechanisms of sense of giving and relationship power. As expected, our results confirmed the proposed chain mediation model in the study. This indicated that sense of giving and relationship power play a crucial bridging role in the effects of love addiction on perceived acceptability of gaslighting.

### 4.1 The influence of love addiction on perceived acceptability of gaslighting

The results indicated that love addiction significantly and positively correlated with perceived acceptability of gaslighting. Individuals with love addiction typically exhibit an insecure attachment style that manifests as dependence and a strong need for validation, which predisposes them to tolerate their partner’s negative behaviors (Henderson et al., 2005; McKeown, 2014; Bonache et al., 2019; Gori et al., 2023). Love addiction may also lead them to reinterpret manipulative and even abusive behaviors as “stress” or “displays of love,” thus turning a blind eye to the harmful effects of these behaviors. This makes it more difficult for them to recognize manipulation, increasing the likelihood that they will be affected by gaslighting (Collins et al., 2002, 2006; Reynaud et al., 2010; Spencer et al., 2021). Another possible factor contributing to this phenomenon is that love addicts may have a pessimistic view of the stability and availability of long-term relationships. As a result, they are more likely to commit to or stay in undesirable relationships, such as unstable or low-quality ones. They may do so even before they are truly ready, fearing that if they do not commit quickly, they might not have another chance for a romantic relationship in the future (Warner and Warner, 2022). This may make similar abusive or violent behavior a normalized phenomenon in their relationships and therefore more acceptable.

Interestingly, the present study demonstrated that love addiction did not directly predict perceived acceptability of gaslighting when sense of giving and relationship power were introduced as mediating variables. This indicates that love addiction functions as a critical distal driver—its influence is fully channeled through the mechanisms of sense of giving and relationship power, rather than operating in isolation. The lack of a direct effect does not diminish its theoretical centrality; instead, it reveals that love addiction initiates a cascade of relational compromises, which culminate in tolerance for gaslighting. Thus, love addiction remains foundational to the model, serving as the root catalyst that activates subsequent unhealthy dynamics.

### 4.2 The mediating role of sense of giving

An important finding of this study is that the sense of giving mediates the relationship between love addiction and perceived acceptability of gaslighting. Research has shown that sacrifice and giving in romantic relationships contribute to relationship quality (Totenhagen et al., 2013; Day and Impett, 2016, 2018). Individuals who are addicted to love likewise invest to run a romantic relationship. While investing in a relationship may lead to temporary relational stability, at the same time the corresponding emotional and psychological costs accumulate, making disengagement increasingly difficult (Goodfriend and Agnew, 2008; Tykocinski and Ortmann, 2011; Xiang et al., 2017). From the perspective of prospect theory, this tendency is consistent with the concept of loss aversion. Love addicts may view their investment in a relationship as a possible loss, and termination of the relationship means that the possible loss is immediately transformed into a definite loss. Risk-seeking motivation due to loss aversion may prompt love addicts to seek ways to continue to keep their romantic relationships growing steadily (Tversky and Kahneman, 1992; Kahneman and Tversky, 2013). Sacrifice and giving in relationships can improve their quality. They help make up for losses and create potential gains. This can lead to a more stable and harmonious relationship. As a result, people invest more in the relationship, and their willingness to give increases (Tykocinski and Ortmann, 2011; Day and Impett, 2016). This aversion to perceived loss reinforces a pattern of tolerance to perceived acceptability of gaslighting in order to avoid the loss of investment and denial of sense of giving associated with relationship breakdown. As the results suggest, love addiction enhances sense of giving, which in turn increases tolerance for gaslighting, illustrating how sense of giving can be a mechanism for self-compromise.

### 4.3 The mediating role of relationship power

The present study also identifies relationship power as a key mediator of the link between love addiction and perceived acceptability of gaslighting. Consistent with previous research, power imbalances are widely recognized as contributing factors to abusive behavior in intimate relationships (Sweet, 2019; Martín-Lanas et al., 2021; Hailes and Goodman, 2023). Previous research suggests that when one partner is overly dependent on a romantic relationship, their ability to exert influence in the relationship may be diminished, leading to a power imbalance (Kelly, 1987; Rusbult and Van Lange, 2003; Lennon et al., 2013). Low-power partners tend to show a “willingness to compromise” in the face of relationship conflict, which may make them more susceptible to manipulation or control by high-power partners (Rusbult et al., 1998; Anderson et al., 2003; Oka et al., 2016; Alonso-Ferres et al., 2021). As this study reveals, people with high levels of love addiction experience diminished relationship power. Excessive dependence and obsession make them more likely to lower their boundaries in romantic relationships, accept unfair relationship terms, and then lose control of the relationship. The power imbalance diminishes their ability to express their personal needs or resist manipulation, ultimately increasing their susceptibility to gaslighting (Drigotas et al., 1999; Sweet, 2019). In this context, love addicts may sacrifice their autonomy to prevent the relationship from breaking down. This, intentionally or unintentionally, reinforces a cycle of dependency, making it easier for their partner to exert control with little resistance. Our findings contribute to a deeper understanding of how relationship power imbalances perpetuate cycles of manipulation.

### 4.4 The chain mediating effect of sense of giving and relationship power

A novel contribution of this study is the identification of a chain mediating effect in which love addiction promotes a stronger sense of giving, which in turn diminishes power in the relationship and increases susceptibility to gaslighting. According to motivation-management theory (Murray and Holmes, 2009), individuals in romantic relationships need to balance two goals: one is to bond with their partner and the other is to protect their interests. To balance these two goals, individuals assess the likelihood of being accepted by their partner. If individuals predict the likelihood of acceptance, they will more actively seek connection with their partner. Whether through acts of giving as a gesture of goodwill or seeking support from a partner, both are proactive ways to build an emotional connection. The goal is to enhance both partners’ evaluations of the relationship and make it more stable. Individuals who value their “highly rated” partner and relationship will promote giving to the partner as a way to justify their commitment to the relationship. This means that the more individuals give to a relationship, the more they will rate that giving and the more they will believe in their love for their partner. However, in the case of love addiction, this balance leans heavily toward connection. Even when individuals feel likely to be rejected, they still tend to sacrifice their own interests, including autonomy, to maintain the relationship. On the other hand, if individuals highly value their investment in a romantic relationship but the relationship fails to progress as desired, they may experience cognitive dissonance. This discomfort can push them to adjust their attitudes or behaviors. According to cognitive dissonance theory (Festinger, 1957), in emotionally charged cognitive dissonance scenarios, individuals high in love addiction may first choose to doubt themselves and further increase their commitment and accept an unequal romantic contract to maintain the romantic relationship. Specifically, highly love-addicted individuals may opt to continue investing substantial resources and further strengthen their commitment to the relationship. Their goal is to strive for its maintenance, despite being fully aware that its future is highly uncertain, or even that it has a low probability of success. The high investments associated with giving, sacrificing, and compromising to the relationship and the expectation of corresponding rewards can gradually reduce an individual’s autonomy and weaken their relationship power. In this case, the sense of giving and compromising not only serves as a mechanism for maintaining the relationship but justifies continued investment within the context of harmful dynamics. This process further diminishes relationship power and increases the individual’s tolerance for gaslighting.

Notably, while cognitive dissonance may be a factor in tolerating gaslighting, it may also incentivize individuals to seek out healthier relationship dynamics, especially in the presence of external support systems. For example, previous research has shown that individuals with supportive resources (e.g., social support) are more likely to confront manipulation and reduce their tolerance of abuse (Dutton and Painter, 1993; Dias et al., 2019). This difference highlights the importance of social support, suggesting that interventions focused on enhancing social resources may help relationship addicts recognize and resist gaslighting.

## 5 Implications and limitations

This study constructed a chain-mediated model of the relationship between love addiction and perceived acceptability of gaslighting. Current research has not yet explored the internal mechanisms of how love addiction affects perceived acceptability of gaslighting. The present study elucidates the mechanisms through which love addiction predisposes individuals to tolerate gaslighting, with critical implications for both theory and practice. Theoretically, our findings advance the literature in three key ways. First, by integrating social exchange and interdependence theories, we unveil a relational cascade (love addiction → sense of giving → relationship power → perceived acceptability of gaslighting) that explains how pathological attachment patterns translate into manipulation tolerance. Unlike prior studies that focused on bivariate associations (Graves and Samp, 2021; Johnson et al., 2021; Klein et al., 2023), our model bridges this gap, offering a unified framework for understanding systemic relational dysfunction. Second, we redefine love addiction as a distal driver of perceived acceptability of gaslighting, aligning with attachment theory’s emphasis on maladaptive relational schemas (Bonache et al., 2019). This conceptual shift urges researchers to move beyond symptom reduction (e.g., treating addiction alone) and instead target the relational processes that sustain manipulation. Third, we advocate for mechanistic models in intimate partner violence research, challenging oversimplified narratives of causality.

Practically, these insights offer actionable pathways for intervention: (1) Reframe cognitive distortions about love and sacrifice (e.g., challenging beliefs that “suffering proves devotion”) through cognitive-behavioral techniques. (2) Restore relational autonomy via empowerment training, such as assertiveness exercises and social support network building, to counteract power imbalances. (3) Implement early screening for love addiction in couples therapy to identify high-risk individuals before gaslighting becomes entrenched. On a broader scale, educational programs promoting healthy relational norms (e.g., reciprocity, boundary-setting) in schools may prevent gaslighting by addressing its root causes. These strategies align with the preventive-intervention framework (Dutton and Painter, 1993), which emphasizes disrupting pathological cycles at multiple levels.

Finally, this study has some limitations: (1) The cross-sectional design limits the ability to establish causal relationships. Future longitudinal studies could provide insights into the temporal dynamics between love addiction, sense of giving, relationship power, and perceived acceptability of gaslighting. (2) This study utilized a questionnaire approach; future research could employ a rigorous experimental research paradigm to validate the underlying mechanisms of how love addiction affects perceived acceptability of gaslighting. (3) This study relies on one-sided self-reports, limiting the ability to capture dyadic interactions in romantic relationships. Future research should collect data from both partners to assess actual gaslighting behaviors, not just perceived of acceptability. Additionally, future studies using actor-partner interdependence modeling (APIM) would provide a more accurate understanding of how partners influence each other. (4) This study only explored how love addiction affects perceived acceptability of gaslighting. Future research could introduce moderating variables (e.g., self-esteem, social support and gender) to examine differences in the impact of love addiction on perceived acceptability of gaslighting across contexts. (5) The sample structure of this study may be biased. Most participants were university students. Future studies could use a broader sample type.

## 6 Conclusion

Based on the findings of the present study, we conclude that love addiction fosters an increased susceptibility to gaslighting through sense of giving and relationship power. By illustrating the independent and chain mediating effects of these factors, this research offers a more nuanced view of the psychological mechanisms that underlie gaslighting in romantic relationships.

## Data Availability

The raw data supporting the conclusions of this article will be made available by the authors, without undue reservation.
